# Modifying the Substrate Specificity of *Carcinoscorpius rotundicauda* Serine Protease Inhibitor Domain 1 to Target Thrombin

**DOI:** 10.1371/journal.pone.0015258

**Published:** 2010-12-20

**Authors:** Pankaj Kumar Giri, Xuhua Tang, Saravanan Thangamani, Rajesh T. Shenoy, Jeak Ling Ding, Kunchithapadam Swaminathan, J. Sivaraman

**Affiliations:** Department of Biological Sciences, National University of Singapore, Singapore, Singapore; University of South Florida College of Medicine, United States of America

## Abstract

Protease inhibitors play a decisive role in maintaining homeostasis and eliciting antimicrobial activities. Invertebrates like the horseshoe crab have developed unique modalities with serine protease inhibitors to detect and respond to microbial and host proteases. Two isoforms of an immunomodulatory two-domain Kazal-like serine protease inhibitor, CrSPI-1 and CrSPI-2, have been recently identified in the hepatopancreas of the horseshoe crab, *Carcinoscorpius rotundicauda*. Full length and domain 2 of CrSPI-1 display powerful inhibitory activities against subtilisin. However, the structure and function of CrSPI-1 domain-1 (D1) remain unknown. Here, we report the crystal structure of CrSPI-1-D1 refined up to 2.0 Å resolution. Despite the close structural homology of CrSPI-1-D1 to rhodniin-D1 (a known thrombin inhibitor), the CrSPI-1-D1 does not inhibit thrombin. This prompted us to modify the selectivity of CrSPI-1-D1 specifically towards thrombin. We illustrate the use of structural information of CrSPI-1-D1 to modify this domain into a potent thrombin inhibitor with IC_50_ of 26.3 nM. In addition, these studies demonstrate that, besides the rigid conformation of the reactive site loop of the inhibitor, the sequence is the most important determinant of the specificity of the inhibitor. This study will lead to the significant application to modify a multi-domain inhibitor protein to target several proteases.

## Introduction

The innate immune system is the first line of inducible host defense against various pathogens and their products [1]. Secreted proteases serve important roles in pathogen virulence. Several families of protease inhibitors from the host play an important role in innate immunity by inactivating and clearing the proteases from the pathogens. Horseshoe crab hemocytes contain granules filled with several serine protease zymogens. During mechanical injury or pathogen invasion, the granules are released into the extracellular milieu by exocytosis, and precursor forms of clotting enzymes are activated by a serine protease cascade triggered by bacterial endotoxin. This pathogen-induced cascade is regulated by three serpins, also known as *Limulus* intracellular coagulation inhibitors (LICI-1, LICI-2 and LICI-3) [2], [3], [4], [5]. Protease inhibitors, thus plays multiple roles by maintaining homeostasis and eliciting innate immunity [6]. This defense system is essential for the survival and perpetuation of all multicellular organisms [6], [7].

The Kazal family is one amongst 18 families of serine protease inhibitors, and is mainly divided into two groups: the classical and the non-classical inhibitors. Non-classical Kazal inhibitors [8] consist of one to seven repeated domains, with each domain constituting 50–60 amino acid residues. Regardless of whether a domain is functionally active, it contains a reactive site loop (RSL) exposed at the surface. The serine protease inhibitor functions as a substrate analogue, but the resulting enzyme-inhibitor complex is very stable [9].

We recently reported a two-domain non-classical Kazal serine protease inhibitor from the hepatopancreas of *Carcinoscorpious rotundicauda* (CrSPI) with a possible dual function of inactivating pathogen protease (subtilisin) and host protease (furins). The full length and domain 2 of CrSPI-1 have been shown to contain full inhibitory activities against subtilisin. However, the function of the domain 1 of CrSPI (hereafter referred to as CrSPI-1-D1) is not yet characterized [10]. Analysis of the CrSPI-1-D1 sequence shows that it is significantly homologous to that of rhodniin-D1 from *Rhodnius prolixus*, which is a thrombin inhibitor [11]. A number of endogenous thrombin inhibitors are available, and the most potent one is hirudin from the medicinal leech, *Hirudo medicinalis* [12].

In spite of several studies on serine protease inhibitors, CrSPIs are relatively new and potent [10]. There are several unexplored potentials and unanswered questions about CrSPIs, for example, what is the structural homology of the CrSPI domains, among themselves and other SPIs? What is the variance of target specificity and inhibition? In order to address these questions we have undertaken the structural and functional studies on CrSPI-1-D1.

Here, we report the crystal structure of CrSPI-1-D1 refined up to 2.0 Å resolution. Despite the close structural homology of CrSPI-1-D1 to rhodniin-D1, the native CrSPI-1-D1 does not inhibit thrombin. This motivated us to modify the selectivity of the CrSPI-1-D1 to specifically target thrombin. We show that sequential mutations in the RSL region of CrSPI-1-D1 generated a potent and specific thrombin inhibitor. The full length CrSPI-1 with this modified role of CrSPI-1-D1 as a thrombin modulator, might play a central role in regulating not only hemostasis but also inflammation, and may provide a close link between these processes and how they might co-evolve in the biological system. Furthermore, the possibilities to further develop this D1 mutant into a shorter yet active anti-thrombin holds potentials for biomedical applications as a coagulation modulator [13], [14], [15], [16], [17], [18], [19], [20].

## Results

### Overall structure

**Figure 1:**
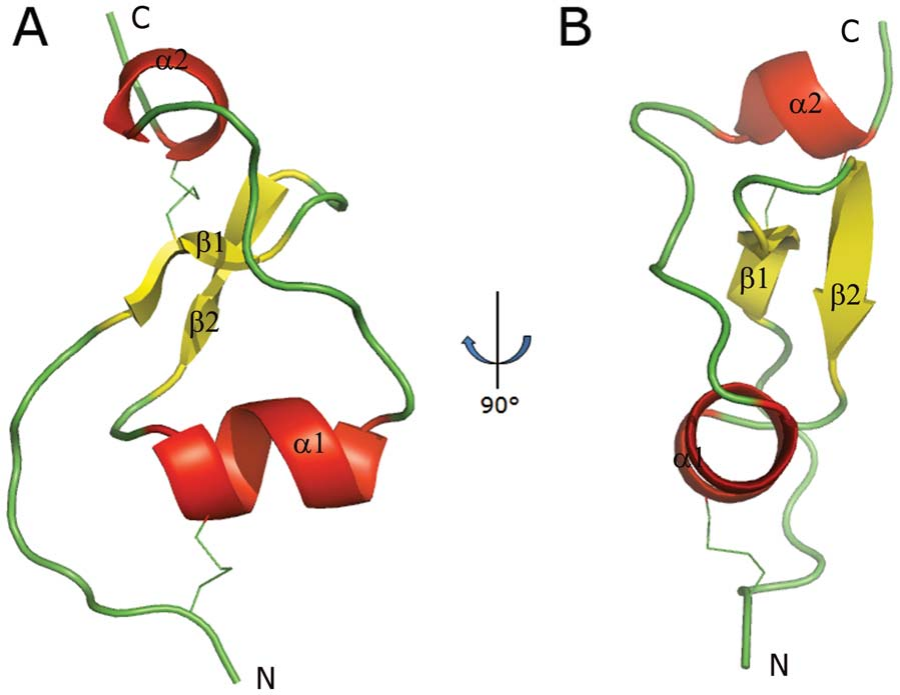
Structure of CrSPI-1-D1. *(A)* Ribbon diagram of the CrSPI-1-D1. *(B)* 90° rotated side view. α-Helix, β-strands and random coils are depicted in red, yellow and green, respectively. The disulfide bridges are shown in green. The secondary structures, N- and C-termini, are labeled. This figure and the following figures of this manuscript were prepared using the program PyMOL[31].

The structure of CrSPI-1-D1 was solved by molecular replacement method and refined to a final R-factor of 0.21 (R_free_ = 0.25) at 2.0 Å resolution. The model has been refined with good stereo chemical parameters (Table 1). There are two CrSPI-1-D1 monomers in the asymmetric unit. The structure of CrSPI-1-D1 mostly consists of loops with a two-strand (Val8-Gly10 and Gly13-Tyr16) β-sheet and a two-turn α-helix (Figure 1). In addition, a single turn α-helix (Trp33-Cys36) is present at the C-terminal. A disulphide bond is located between Cys1 and Cys20 to help maintain the rigidity of the RSL. The carboxyl terminus is linked to the N-terminal through a second disulphide bridge, Cys9-Cys36 (Figure 1A).

**Table 1:** Data collection and refinement statistics of CrSPI-1-D1.

***Experiment***	
Cell parameters (Å, °)	a = 25.5, b = 37.2, c = 36.5, α = 90, β = 99.8, γ = 90
Space group	P2_1_
***Data collection*** a	
Resolution range (Å)	50.0-2.0 (2.07-2.00)
Observed Reflections	28,930
Unique Reflections	4,535
Redundancy	6.4 (3.2)
Completeness (%)	97.3 (80.7)
Overall (*I/σI)*	31.3
Rsym^b^	0.046 (0.061)
***Refinement***	
Resolution range (Å)	20.0-2.0
Number of Reflections used	4432
R factor^c^/R_free_ d (%)	21.47/25.56
RMSD bond lengths (Å)	0.009
RMSD bond angles (°)	1.660
Average B-factors (Å^2^)	18.046
Main chain (# atoms)	16.045 (312)
Side chain (# atoms)	18.394 (264)
Water (# atoms)	27.559 (56)
***Ramachandran Plot***	
Most favored region (%)	93.4
Additional allowed regions (%)	4.9
Generously allowed regions (%)	1.6
Disallowed regions (%)	0.0

a Numbers in parentheses refer to the highest resolution shell.

b R_sym_  =  Σ|*I_i_* – <*I_i_>*|/Σ| *I_i_|.*

c R_factor_  =  Σ||F_obs_| − |F_calc_||/Σ|F_obs_|.

d R_free_ equals the R factor against 5.9% of the data removed prior to refinement.

### Structural comparison

**Figure 2:**
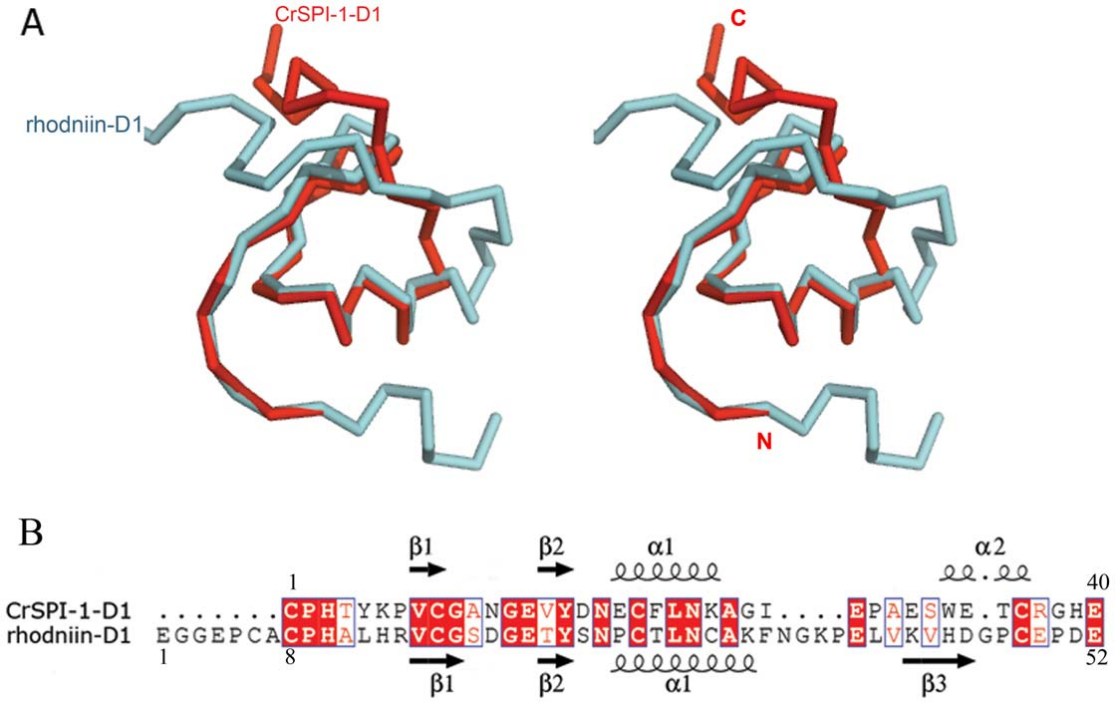
Comparison of CrSPI-1-D1 with rhodniin-D1. *(A)* Stereo Cα superposition of CrSPI-1-D1 (red) and rhodniin-D1 (cyan). The RMSD between CrSPI-1-D1 and rhodniin-D1 is 2.0 Å for 36 Cα atoms. *(B)* Structure based sequence alignment between CrSPI-1-D1 and rhodniin-D1. This alignment was performed using the program COOT [28]. The secondary structural elements for CrSPI-1-D1 and rhodniin-D1 are shown at the top and the bottom, respectively. The conserved residues are highlighted in red boxes outlined in blue. This figure was created by using the program ESPript [32].

A search for topologically similar domains within the PDB database using the DALI program [21] revealed that the structural features of CrSPI-1-D1 resemble the typical non-classical Kazal type inhibitor [8]. The highest structural similarity is observed between hirudin, the leech-derived tryptase inhibitor from *H. medicinalis* and CrSPI-1-D1, yielding an rmsd of 1.9 Å for 36 Cα atoms (pdb code 1ldt). This is followed by a thrombin protease inhibitor, rhodniin domain 1 (rhodniin-D1) from *Rhodnius prolixus,* which yielded an rmsd of 2.0 Å for 36 Cα atoms (pdb code 1tbq). In addition to the structural homology, the CrSPI-1-D1 and rhodniin-D1 display 42% sequence identity while only 35% sequence identity was observed with hirudin. The structure-based sequence alignment revealed that most of the structurally invariant residues are located at the carboxy terminus, including the RSL, β1, β2 and α1 of CrSPI-1-D1 (Figure 2). These observed features provided a clue that CrSPI-1-D1 might specifically target thrombin after modifications of a few residues in the RSL, and this prompted us to change the specificity of CrSPI-1-D1 to target thrombin.

### The reactive-site loop

**Figure 3:**
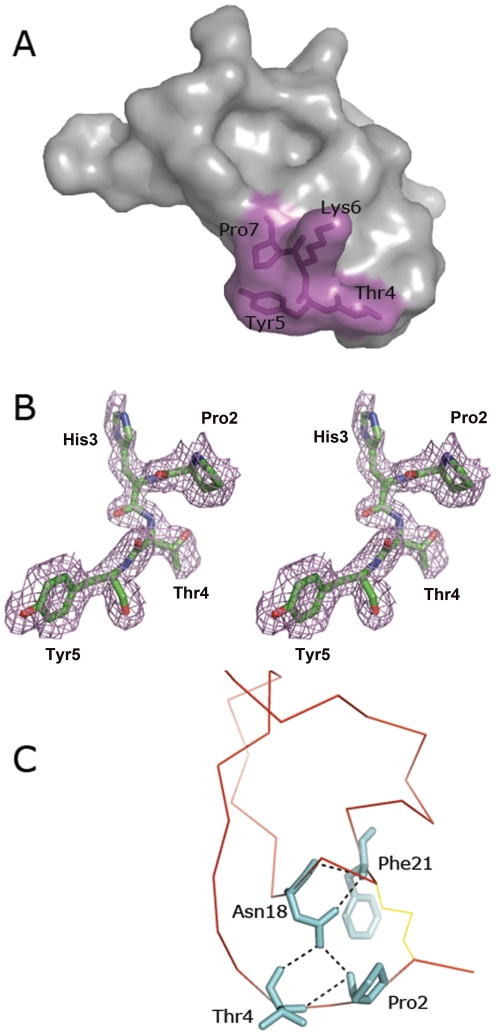
The reactive-site loop (RSL). *(A)* The site-directed mutation on CrSPI-1-D1. A transparent surface representation of the CrSPI-1-D1 is shown with all mutated residues in stick representation in magenta. *(B)* Stereo view of the electron density map. Simulated annealing *Fo-Fc* omit map of the N-terminal region of CrSPI-1-D1 showing the key residues in reactive-site loop. All residues shown in this figure as well as all atoms within 2 Å of these residues were omitted prior to refinement and map calculation. The map was contoured at a level of 2.0 σ. *(C)* A close view shows the interactions aid in maintaining the rigidity of the RSL. Cα of the CrSPI-1-D1 is shown in red. The disulfide bonds are shown in yellow, stick line while the hydrogen bonds are shown in black, dotted lines.

Although the sequence of the reactive-site loop (RSL) is different in several families of serine protease inhibitors, the conformation of the RSL is similar [10], [11]. Like other Kazal-type inhibitors, the disulfide bonds formed by cysteine residues at the P3 and P5′ positions (Cys1 and Cys9 in CrSPI-1-D1) hold the RSL in a relatively rigid conformation. Besides, there are several internal hydrogen bonds (<3.2 Å) which help maintain the rigidity of the RSL in the CrSPI-1-D1. Figure 3c shows selected hydrogen bonding contacts between RSL and CrSPI-1-D1. Notably, strong intra-molecular H-bonds (<3.0 Å) were observed between the carbonyl oxygen of Pro2 (P2 position) and amide nitrogen of Thr4 (P1′ position); Asn18 and Phe21, ND2 of Asn18 interacts with the main chain carbonyl atoms of Pro2 and Thr4 at the P2 and P1′ positions of the RSL, respectively (Table S1). Similar interactions were observed in rhodniin-D1 and other protease inhibitors such as the turkey ovomucoid third domain, OMTKY3, although there are different amino acids in those positions [22]. In addition to the S-S bonds, these hydrogen bonds are essential to maintain the rigidity of the RSL during the inhibition of the cognate enzyme. Although a similar rigid conformation is found in these inhibitors, they recognize the substrates differently. This clearly shows that in addition to the rigid conformation, the sequence of the RSL dictates the selectivity towards a particular protease. Thus, we have mutated the RSL side chains of CrSPI-1-D1 to specifically target thrombin.

### Mutations to change the specificity

**Figure 4:**
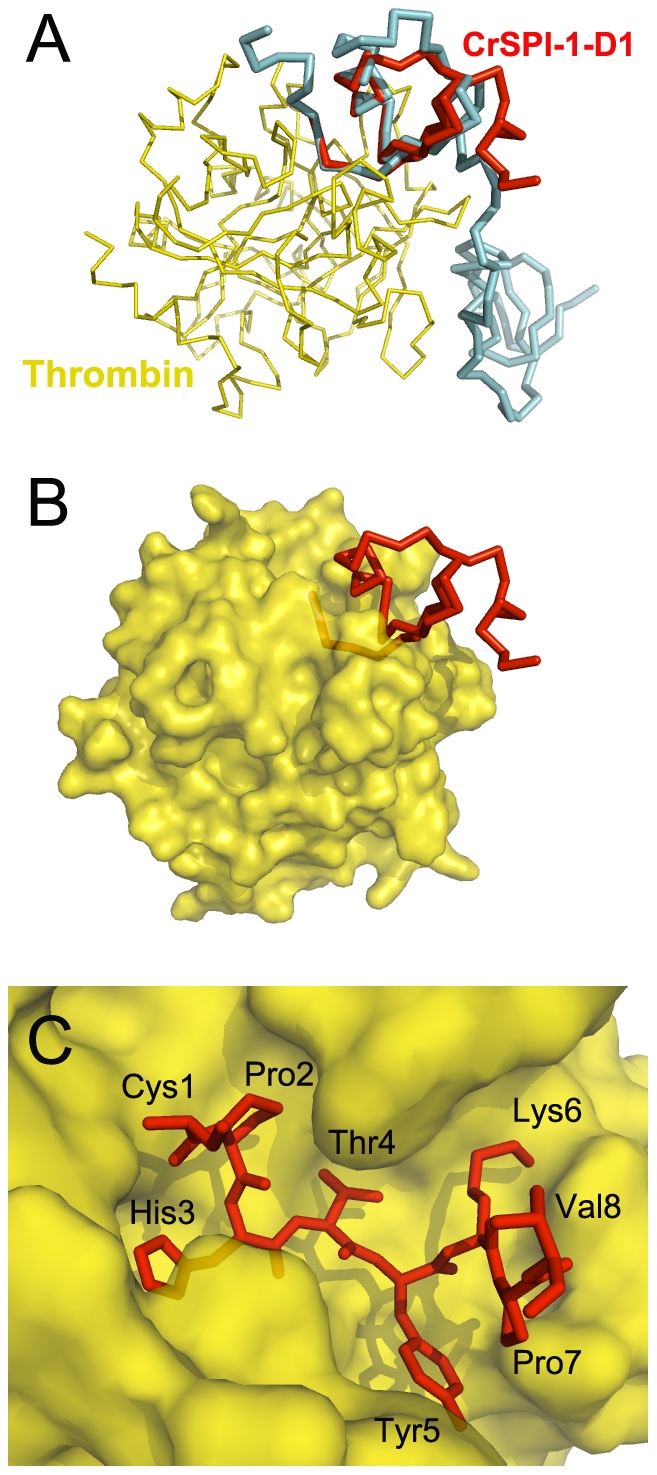
Modeling complex of CrSPI-1-D1 with thrombin. *(A)* Complex structure of rhodniin with thrombin (pdb code 1tbq). Rhodniin and thrombin are shown in cyan and yellow, respectively. CrSPI-1-D1 (red) was superimposed on rhodniin-D1. *(B)* Thrombin is shown as surface representation in this model complex. *(C)* Close up view of the RSL of CrSPI-1-D1 in the active site of thrombin.

Following the structure determination of CrSPI-1-D1, the next main objective was to elucidate the inhibitory efficiency of this domain. Our previous studies showed that full length as well as domain 2 of CrSPI-1 is a specific inhibitor of subtilisin, however the specificity of domain-1 is not yet established [10]. An analysis of P3 to P4′ residues of the RSLs of various substrates like binding serine protease inhibitors such as for subtilisin, thrombin, trypsin, chymotrypsin and furin was performed to identify the minimum side chains of CrSPI-1-D1 to be mutated to alter the selectivity (Table 2). The closest similarities were observed with RSLs of rhodniin-domain-1. P3, P2 and P1 of CrSPI-1-D1 and rhodniin-D1 are similar, but P1′, P2′, P3′ and P4′ were different. Complex crystal structure of rhodniin and thrombin showed that the N terminal domain of rhodniin interacts with the active-site cleft region of thrombin (PDB 1tbq). In addition to the interactions of Pro9, His10 and Alall, the side chain of Leu12 occupies the S2′ site of thrombin. His13 mediates a hydrogen bond and stacks with aromatic residues in S3′. Arg14 at P4′ allows charge compensation of Glu39 from thrombin. The clustering of the positively charged inhibitor residues at P3′ and P4′ might be particularly beneficial for thrombin binding [23]. Based on rhodniin-D1: thrombin complex structure a model for CrSPI-1-D1: thrombin complex was constructed. This model showed that the RSL of CrSPI-1-D1 fit well in the active site of thrombin (Figure 4). These observations lead to the introduction of mutations in the RSL region of CrSPI-1-D1 (Table S2), which was previously of uncharacterized function, to specifically target thrombin.

**Table 2:** Reactive site loop regions from P3 to P4′ position of selected serine protease inhibitors.

Inhibitors	P3	P2	P1	P1′	P2′	P3′	P4′	Inhibitor against	[ref.]
CrSPI-1 domain-1	Cys1	Pro2	His3	Thr4	Tyr5	Lys6	Pro7	Uncharac--terized	[10]
CrSPI-1 domain-2	Cys46	Thr47	Glu48	Glu49	Tyr50	Asp51	Pro52	Subtilisin	[10]
rhodniin domain-1	Cys8	Pro9	His10	Ala11	Leu12	His13	Arg14	Thrombin	[11]
rhodniin domain-2	Asp61	Gly62	Asp63	Glu64	Tyr65	Lys66	Pro67	Thrombin	[11]
LDTI (Leech Derived Tryptase Inhibitor)	Cys6	Pro7	Lys8	Ile9	Leu10	Lys11	Pro11	Trypsin	[33]
Tomato inhibitor II domain-1	Cys3	Thr4	Arg5	Glu6	Cys7	Gly8	Asn9	Subtilisin	[34]
Tomato inhibitor II dommain-2	Cys60	Thr61	Phe62	Asn63	Cys64	Asp65	Pro66	Subtilisin	[34]
OMTKY3	Cys16	Thr17	Leu18	Glu19	Tyr20	Arg21	Pro22	Subtilisin	[22]
Spn4A (Furin inhibitorfrom Drosophila)	Arg371	Lys372	Arg373	Ala374	Ile375	Met376	Ser377	Furin	[35]
Human PI8(Furin Inhibitor)	Asn337	Ser338	Arg339	Cys340	Ser341	Arg342	Met343	Furin	[36]

Our approach was to mimic the P1′, P2′, P3′ and P4′ residues (Thr4, Tyr5, Lys6 and Pro7) of CrSPI-1-D1 to rhodniin-D1 (Ala11, Leu12, His13 and Arg14); to sequentially mutate and evaluate the implication of these four residues towards thrombin inhibition. In addition to the tetra mutant, we have tried all possible single, double and triple mutants. A total of 15 mutants (Table 3) have been generated and their thrombin inhibition was studied. All the mutants were expressed in bacteria and purified as wild type CrSPI-1-D1 (Figure S1).The CD spectrum was recorded on all 15 mutants of CrSPI-1-D1, which indicated that these mutants share the same α/β structure as the wild type CrSPI-1-D1 (Figure S2). Furthermore, the ESI-MS spectrum showed their expected molecular mass (Figure S3).

**Table 3:** IC_50_ and dissociation constant (K_d_) for the inhibition of thrombin by various variants of CrSPI-1-D1.

S.No.	Mutants	IC_50_	K_d_ (dissociation Const.)
1	T4A	ND	ND
2	Y5K	ND	ND
3	K6H	ND	ND
4	P7R	ND	ND
5	T4A,Y5K	ND	ND
6	T4A,K6H	ND	ND
7	T4A,P7R	ND	ND
8	Y5K,K6H	ND	ND
9	Y5K,P7R	ND	ND
10	K6H,P7R	ND	ND
11	T4A,Y5K,K6H	ND	ND
12	T4A,Y5K,P7R	ND	ND
13	T4A,K6H,P7R	ND	ND
14	Y5K,K6H,P7R	ND	ND
15	T4A,Y5K,K6H,P7R	26.3 nM	4 µM

**ND- not detected.**

We have verified the stability of the CrSPI-1-D1 mutants as a possible inhibitor against different serine proteases such as thrombin, trypsin, chymotrypsin, elastase and subtilisin. Notably only the tetra mutant is stable against thrombin, whereas other serine proteases degrade the modified CrSPI-1-D1, which seemed to act more as a substrate rather than an inhibitor (Figure S4). It suggests that CrSPI-1-D1 mutant is thrombin-specific. In the following section, we describe the inhibition studies of CrSPI-1-D1 mutants with thrombin.

### Thrombin inhibition assay

Previously it has been shown that hirudin has very high inhibitory activity against the human α-thrombin [24]. We chose to study the properties of these CrSPI-1-D1 variants under a similar condition as hirudin: human α- thrombin complex. Out of all 15 CrSPI-1-D1 mutants, only tetra mutant (T4A, Y5K, K6H, and P7R) showed the highest significant inhibition with human α- thrombin in a dose-dependent manner.

**Figure 5:**
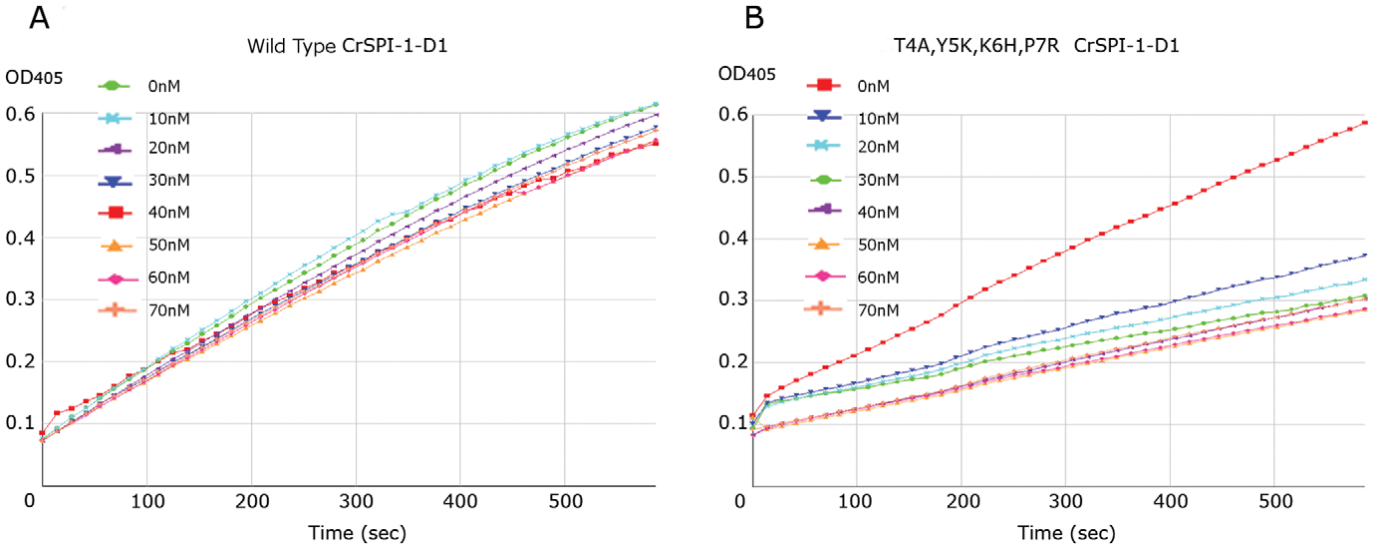
Inhibition of human α-thrombin by increasing concentration of: *(A)* wild type CrSPI-1-D1 and *(B)* T4A, Y5K, K6H, P7R CrSPI-1-D1. Residual protease activity was measured as OD_405_ based on hydrolysis of 0.1 mM S2238 (H-D-Phenylalanyl-L-pipecolyl-Larginine-p-nitroaniline dihydrochloride) and formation of colored product, p-nitroaniline, by 4.5 nM thrombin in a total reaction volume of 200 µL. The concentration of wild type CrSPI-1-D1 and T4A, Y5K, K6H, P7R CrSPI-1-D1 are indicated next to the curve.

**Figure 6:**
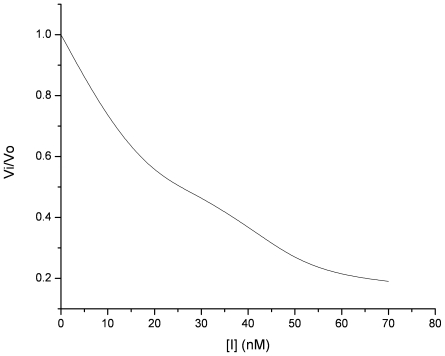
Determination of IC_50_ values based on dose response plots of fractional velocity as a function of different tetra mutant CrSPI-1-D1 concentration. OD_405_ was monitored based on hydrolysis of 0.1 mM S2238 (H-D-Phenylalanyl-L-pipecolyl-Larginine-p-nitroaniline dihydrochloride) and formation of colored product, p-nitroaniline, by 4.5 nM thrombin in a total reaction volume of 200 µL. V_0_ and V_i_ are the initial velocities in the absence and presence of tetra mutant CrSPI-1-D1, respectively and were calculated from Beer-Lambert's Law. The tetra mutant CrSPI-1-D1 concentration predicted to block 50% of the activity of a fixed concentration of thrombin was obtained on a graphical plot of V_i_/V_0_ versus inhibitor concentration.

Figure 5 shows the typical dose-response curves. Wild type CrSPI-1-D1 showed no inhibition, whereas the tetra mutant exhibited strong inhibition against thrombin. The dose response plot of the fractional velocity as a function of different concentrations of tetra mutant CrSPI-1-D1 showed that 26.3 nM of tetra mutant CrSPI-1-D1 was sufficient to inhibit 50% of 4.5 nM thrombin (Figure 6). Since the IC_50_ value of 26.3 nM is within a factor of 10 of the concentration of thrombin and CrSPI-1-D1, it is ascertained that the mode of inhibition follows the typical kazal domain's mode of inhibition. Following the inhibition studies, we verified the binding affinities of these mutants of CrSPI-1-D1 with human α- thrombin using ITC experiments.

### Isothermal Titration Calorimetry (ITC) studies

**Figure 7:**
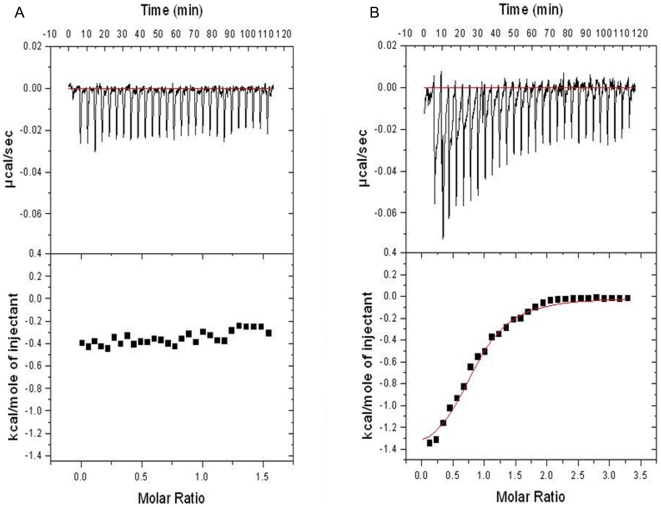
Isothermal Titration Calorimetry analysis. *(A)* Human α- thrombin- wild type CrSPI-1-D1 titration. *(B)* Human α- thrombin - T4A, Y5K, K6H, P7R CrSPI-1-D1 titration. The upper panels show the injection profile after baseline correction and the bottom panel shows the integration (heat release) for each injection.

To verify the interactions between the CrSPI-1-D1 and thrombin, we have performed ITC experiments with wild type CrSPI-1-D1 and selected mutants against thrombin. The wild type CrSPI-1-D1 and the mutants which lacked thrombin inhibition did not show any binding with thrombin. Consistent with the results of thrombin inhibition assays, only the tetra mutant showed interactions with human α- thrombin with dissociation constant (Kd) of 4 µM (Figure 7). The model used for the ITC analysis is a single site binding model assuming a stoichiometric ratio of 1∶1 (CrSPI-1-D1: thrombin).

## Discussion

The *Carcinoscorpius rotundicauda* is an ancient invertebrate that has survived for several hundred million years, and thus termed a ‘living fossil’. Being able to efficiently defend against the multitude of pathogens that thrive in its habitat and survive in this harsh environment, suggests that it possesses a very powerful innate immune defense system. Serine Protease Inhibitors (SPIs) serve important roles in immunity by inactivating and clearing the proteases from the invading pathogens, which use them as virulence factors. How did multidomain SPIs arise? The SPI domains are ‘evolutionarily mobile’ [25]. In the process of evolution, domains from different families of SPIs could have been shuffled and fused in a single inhibitor, resulting in a multidomain inhibitor.

The evolutionary mechanisms of SPIs serve to increase their variety and expand their functions, thus helping to meet the demands of the repertoire of endogenous and exogenous SPs an organism encounters. Thus, knowing the structure of an inhibitor usually provides insights into its inhibitory functions. More importantly, the structural changes of a protease inhibitor in complex with its target protease can provide useful information on the interaction between the two proteins, thus allowing the development of analogs of that inhibitor with increased affinity towards the protease to achieve greater inhibition capacity. This motivated us to modify the selectivity of CrSPI-1-D1 to specifically target thrombin and here we show that selected mutation in the RSL region of CrSPI-1-D1 led to a potent and specific thrombin inhibitor.

We have determined the crystal structure of CrSPI-1-D1 refined up to 2.0 Å resolution, from the horseshoe crab, *C. rotundicauda*. Although the native CrSPI-1-D1 itself is highly homologous to the thrombin inhibitor, rhodniin domain 1 (rhodniin-D1), native CrSPI-1-D1 does not inhibit thrombin. Therefore, our site directed mutation of the RSL represents a structure-based drug design approach in the conversion of an uncharacterized CrSPI-1-D1 into a potent thrombin inhibitor with an IC_50_ of 26.3 nM. Furthermore, our studies revealed that besides the rigid conformation of the RSL, the sequence is most important in dictating the specificity of the inhibitor. This study adds an important implication to modifying a multidomain inhibitor protein. The CrSPI-1 has been shown to target two molecules of proteases. The modified domain D1 targets thrombin, whereas the wild type domain D2 targets subtilisin ([10]; Rajesh TS unpublished data). Moreover, this may lead to further development of the D1 mutant into a shorter active anti-thrombin inhibitor for therapeutic interventions.

## Materials and Methods

### Plasmid and strain construction

The CrSPI-1-D1 (encoding Cys1-Glu40) was PCR amplified using forward CTACTGGATCCTGTCCTCAT and reverse GCAGAGTTCGAATTCCTAGCAAGTTTCCCA primers that were designed to introduce a *Bam* H1 site to the 5′ end and an *Eco* R1 site to the 3′ end. Such PCR fragments were then digested with *Bam* H1 and *Eco* R1, and ligated into pET-M vector, which were previously linearized by compatible restriction enzymes, and transformed into *Escherichia coli,* BL 21.

### Purification

Optimal expression of the CrSPI-1-D1 in bacteria was obtained by induction with 0.5 mM Isopropyl β-D-1-thiogalactopyranoside (IPTG) of 1 liter culture at 25°C. The cells were then disrupted by French Press and the supernatant were collected after centrifuging at 10,000x g for 1 h at 4°C. His-tagged CrSPI-1-D1 proteins were purified in two steps using Ni-NTA (Qiagen) affinity chromatography followed by a Superdex 75 gel filtration column on the Äkta Express (GE Healthcare). The buffer was exchanged to a solution containing 20 mM Tris (pH-8.5), 150 mM NaCl, 5 mM dithiothreitol (DTT) and finally concentrated up to 10 mg/ml.

### Crystallization and structure determination

Initial crystallization conditions were screened at 25°C in the hanging drop vapor diffusion technique using Hampton Research crystallization screens and JB crystallization screens (Jena Biosciences) with drops containing equal volumes (1 µl) of reservoir and protein solution of 10 mg/ml against 0.5 ml of reservoir. Small rod-shaped crystals were formed within 2–3 days. Further optimization by equilibrating 1 µl CrSPI-1-D1 protein solution of 15 mg/ml and 1 µl reservoir solution (0.4 M mono ammonium dihydrogen sulphate, 0.1 M Tris-HCl pH 8.5) using hanging drop vapor diffusion technique at 20°C led to best diffraction-quality crystals. The crystals diffracted up to 2.0 Å and belonged to space group P2_1_ with solvent content is approximately of 35% (Vm  = 1.9 Å^3^/kDa).

Crystals were cryo-protected in the reservoir solution supplemented with 25–30% glycerol, and flash cooled at 100 K. The diffraction data were obtained using a CCD detector (Platinum 135) mounted on a Bruker Microstar Ultra rotating anode generator (Bruker AXS, Madison, WI). All datasets were processed with HKL2000 [26]. The structures were solved by molecular replacement with PHASER [27]. Subsequently the models were manually built by using COOT [28], followed by refinement using CNS [29]. The data collection and refinement statistics are provided in Table 1.

### Site-directed mutagenesis

Based on the rhodniin-thrombin complex structure (PDB code 1tbq), residues Thr4, Tyr5, Lys6 and Pro7 of CrSPI-1-D1 were mutated to Ala, Leu, His and Arg respectively. These are the corresponding residues 8-11 of rhodniin that are crucial for interaction with thrombin (Table S2). We used inverse PCR based mutagenesis [30] to generate all mutants. In total, we generated 15 mutants (single to tetra). All mutant inhibitor proteins were expressed in *E. coli* (BL21DE3) using optimized expression conditions and purified by His-tag based affinity and size exclusion column chromatography. Further the purified CrSPI1-D1 was passed through the reverse phase chromatography using an analytical Jupiter C18 column. The molecular masses of the RP-HPLC purified mutants were determined by ESI-MS on a Perkin-Elmer Sciex API III triple-stage quadrupole instrument equipped with an ionspray interface.

### CD spectroscopy

Far-UV CD spectra (260–190 nm) of CrSPI-1-D1 dissolved in 20 mM Tris-HCl buffer (pH 7.4) at a 30 µM protein concentration were collected using a Jasco J-810 spectropolarimeter (Easton, MD). All measurements were carried out at room temperature using 0.1-cm path length cuvettes with a scan speed of 50 nm/min, a resolution of 0.2 nm, and a bandwidth of 2 nm.

### Stability verification of CrSPI-1-D1 mutants against serine proteases

20 µL of 1 mg/ml CrSPI-1-D1 mutants were incubated with 1 µL of 1 mg/ml of different serine proteases such as thrombin, trypsin, chymotrypsin, elastase and subtilisin at 37°C for 30 minutes. Reaction was stopped by heating the sample with 5X SDS loading dye at 100°C. SDS PAGE was carried out following a standard protocol.

### Inhibition of Thrombin Amidolytic Activity

The buffer used in all functional assays was 20 mM Tris-HCl, pH 7.4. For all thrombin amidolytic activity assay, we used S2238 (H-D-Phenylalanyl-L-pipecolyl-Larginine-p-nitroaniline dihydrochloride), which is a chromogenic substrate for thrombin from Chromogenix (Milano, Italy). To measure the inhibition activity of different CrSPI-1-D1 proteins on thrombin activity, we performed all reactions in 96-wells microtiter plates. For each inhibition assay, 50 µl of 4.5 nM human α- thrombin was pre-incubated for 30 minutes at 37°C with increasing amounts (10 to 70 nM) 50 µl of CrSPI-1-D1 in a total reaction volume of 200 µl, prior to adding 100 µl of S2238. The rate of formation of colored product, p-nitroaniline, was read using an enzyme-linked immunosorbent assay plate reader at 405 nm for 10 minutes. Appropriate negative controls without the thrombin was assayed simultaneously. Percentage inhibition was calculated by taking the rate of increase in absorbance in the absence of inhibitor as 0%. A decrease in absorbance indicated the inhibitory effect of CrSPI-1-D1 on thrombin activity.

### Isothermal Titration Calorimetry (ITC)

The ITC experiments were carried out using VP-ITC calorimeter (Microcal, LLC) at 20°C using 300 µM of the protein in the sample cell and 40 µM of human α-thrombin in the injector. All samples were thoroughly degassed and then centrifuged to get rid of precipitates. Volumes of 10 µl per injection were used for the different experiments. For every experiment, the heat of dilution for each ligand was measured and subtracted from the calorimetric titration experimental 30 runs for the protein. Consecutive injections were separated by at least 4 minutes to allow the peak to return to the baseline. The ITC data was analyzed using a single site fitting model using Origin 7.0 (OriginLab Corp.) software.

### Accession Number

Coordinates of CrSPI-1-D1 have been deposited in the Protein Data Bank (http://www.pdb.org) with accession code **3PIS**.

## Supporting Information

Table S1 **Interactions associated for rigidity of reactive site loop of CrSPI-1-D1.**
(DOC)

Table S2 **Site-directed mutagenesis strategy for CrSPI-1-D1.**
(DOC)

Figure S1 **Reverse Phase-HPLC profile of CrSPI-1-D1**. The purified CrSPI-1-D1 was loaded onto an analytical Jupiter C18 analytical column on SMART Workstation (GE-healthcare) and eluted using a gradient (15 - 40% over 60 min) of buffer B (80% ACN in 0.1% TFA. Figure shows the elution of protein monitored at 215 nm. The peak (indicated with the arrow) contains a single homogenous CrSPI-1-D1 taken for kinetics studies.
(TIF)

Figure S2 **CD spectroscopy profile of reverse phase HPLC purified CrSPI-1-D1**. Far-UV CD spectra (260–190 nm) of CrSPI-1-D1 dissolved in 20 mM Tris-HCl buffer (pH 7.4) at a 30 μM protein concentration were collected using a Jasco J-810 spectropolarimeter (Easton, MD). All measurements were carried out at room temperature using 0.1-cm path length cuvettes with a scan speed of 50 nm/min, a resolution of 0.2 nm, and a bandwidth of 2 nm. The CD spectrum of the tetra mutant of CrSPI-1-D1 indicated that it assumed an α/β structure.
(TIF)

Figure S3 **ESI/MS profile of reverse phase HPLC purified CrSPI-1-D1**. The spectrum shows a series of multiply charged ions, corresponding to the correct molecular mass of 6644± 0.22 Da. The purity and mass of all mutant proteins of CrSPI-1-D1 were determined by electro spray ionization mass spectrometry using an API 300 liquid chromatography tandem mass spectrometry system (PerkinElmer Life Sciences Sciex, Selton, CT).
(TIF)

Figure S4 **The specificity of CrSPI-1-D1 tetra mutant for thrombin ascertained by comparison with other proteases.** SDS-PAGE analysis for the interaction of CrSPI-1-D1 wild type and tetra mutant with different proteases. ***A*** *)* Lane 1 protein marker; Lane 2 CrSPI-1-D1 alone and Lane 3-7 CrSPI-1-D1 wild type incubated with human α-thrombin, chymotrypsin, trypsin, elastase and subtilisin, respectively, for 37°C for 30 minutes. ***B*** *)* Lane 1 protein marker; Lane 2 T4A, Y5K, K6H, P7R CrSPI-1-D1 alone and Lane 3-7 T4A,Y5K, K6H, P7R CrSPI-1-D1 incubated with human α-thrombin, chymotrypsin, trypsin, elastase and subtilisin, respectively, for 37°C for 30 minutes.
(TIF)
