# Supplementary material for: Modifying the Substrate Specificity of Carcinoscorpius rotundicauda Serine Protease Inhibitor Domain 1 to Target Thrombin
Source: PLoS One. 2010 Dec 20;5(12):e15258. doi: 10.1371/journal.pone.0015258 (PMC3004852; doi:10.1371/journal.pone.0015258)
Supplement: Table S1 — Interactions associated for rigidity of reactive site loop of CrSPI-1-D1. (DOC) [file pone.0015258.s001.doc]

**Table S1 Interactions associated for rigidity of reactive site loop of CrSPI-1-D1.**

|  | **Interaction** | **Distance (Ǻ)** |
| --- | --- | --- |
| CrSPI-1-D1 | O (Pro2,P1΄ position)-ND2 (Asn18) | 2.94 |
|  | O (Thr4,P2΄ position)-ND2 (Asn18) | 2.85 |
|  | OD1 (Asn18)- N(Phe21) | 2.86 |
|  | O(Asn18)-N(Phe21) | 3.14 |
|  | O (Pro2,P1΄ position)-N (Thr4,P2 position) | 3.57 |
| rhodniin-D1 | O (Pro9,P1΄ position)-ND2 (Asn25) | 3.50 |
|  | O (Ala 11,P2 position )-ND2 (Asn25) | 2.80 |
|  | OD1 (Asn18)- N(Thr28) | 3.17 |
|  | O(Asn18)-N(Thr28) | 3.13 |
|  | O (Pro9,P1΄ position)-N (Ala 11,P2 position) | 3.25 |
| OMTYK3 | O (Thr 17,P1΄ position)-ND2 (Asn33) | 2.84 |
|  | O (Glu 19,P2 position )-ND2 (Asn33) | 3.00 |
|  | OD1 (Asn33)- N(Asn 36) | 3.00 |
|  | O(Asn18)-N(Asn 36) | 3.20 |
|  | O (Thr17,P1΄ position)-N (Glu19,P2 position) | 3.75 |
