# Supplementary material for: Modifying the Substrate Specificity of Carcinoscorpius rotundicauda Serine Protease Inhibitor Domain 1 to Target Thrombin
Source: PLoS One. 2010 Dec 20;5(12):e15258. doi: 10.1371/journal.pone.0015258 (PMC3004852; doi:10.1371/journal.pone.0015258)
Supplement: Table S2 — Site-directed mutagenesis strategy for CrSPI-1-D1. (DOC) [file pone.0015258.s002.doc]

**Table S2 Site-directed mutagenesis strategy for CrSPI-1-D1.**

| S.No. | Rhodniin | CrSPI-1-D1 | |
| --- | --- | --- | --- |
|  |  | Wild type | after mutation |
| 1 | 8ALA**(GCT)** | 4Thr(ACT) | 4ALA(GCT) |
| 2 | 9Leu**(CTG)** | 5Tyr(TAC) | 5Leu(TTG) |
| 3 | 10His (CAT) | 6Lys(AAA) | 6His (CAT) |
| 4 | 11Arg (AGA) | 7Pro(CCT) | 7Arg (CGT) |
