# Supplementary figures and images for: Modifying the Substrate Specificity of Carcinoscorpius rotundicauda Serine Protease Inhibitor Domain 1 to Target Thrombin
Source: PLoS One. 2010 Dec 20;5(12):e15258. doi: 10.1371/journal.pone.0015258 (PMC3004852; doi:10.1371/journal.pone.0015258)

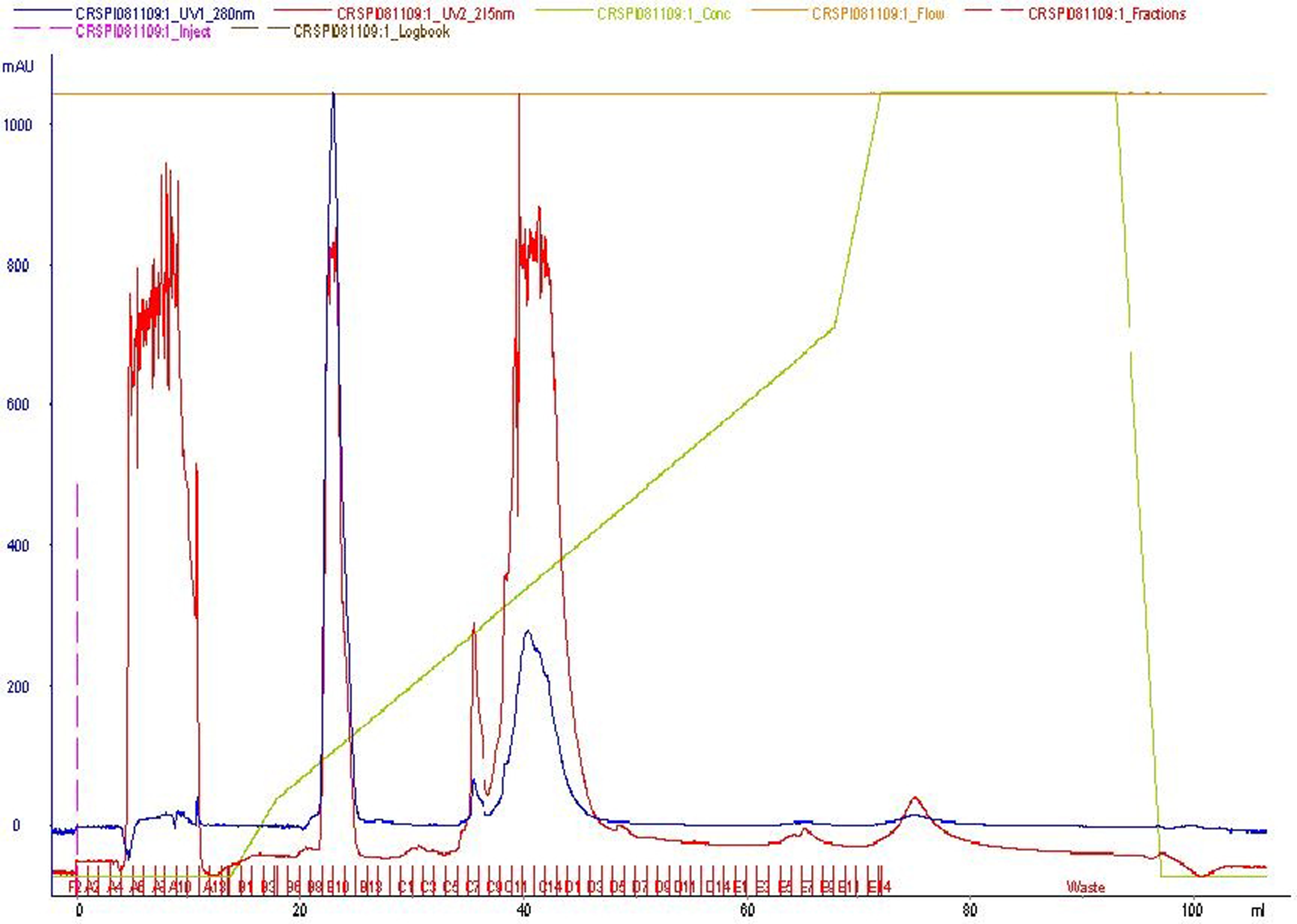

Supplement: Figure S1 — Reverse Phase-HPLC profile of CrSPI-1-D1. The purified CrSPI-1-D1 was loaded onto an analytical Jupiter C18 analytical column on SMART Workstation (GE-healthcare) and eluted using a gradient (15 - 40% over 60 min) of buffer B (80% ACN in 0.1% TFA. Figure shows the elution of protein monitored at 215 nm. The peak (indicated with the arrow) contains a single homogenous CrSPI-1-D1 taken for kinetics studies. (TIF) [file pone.0015258.s003.tif]

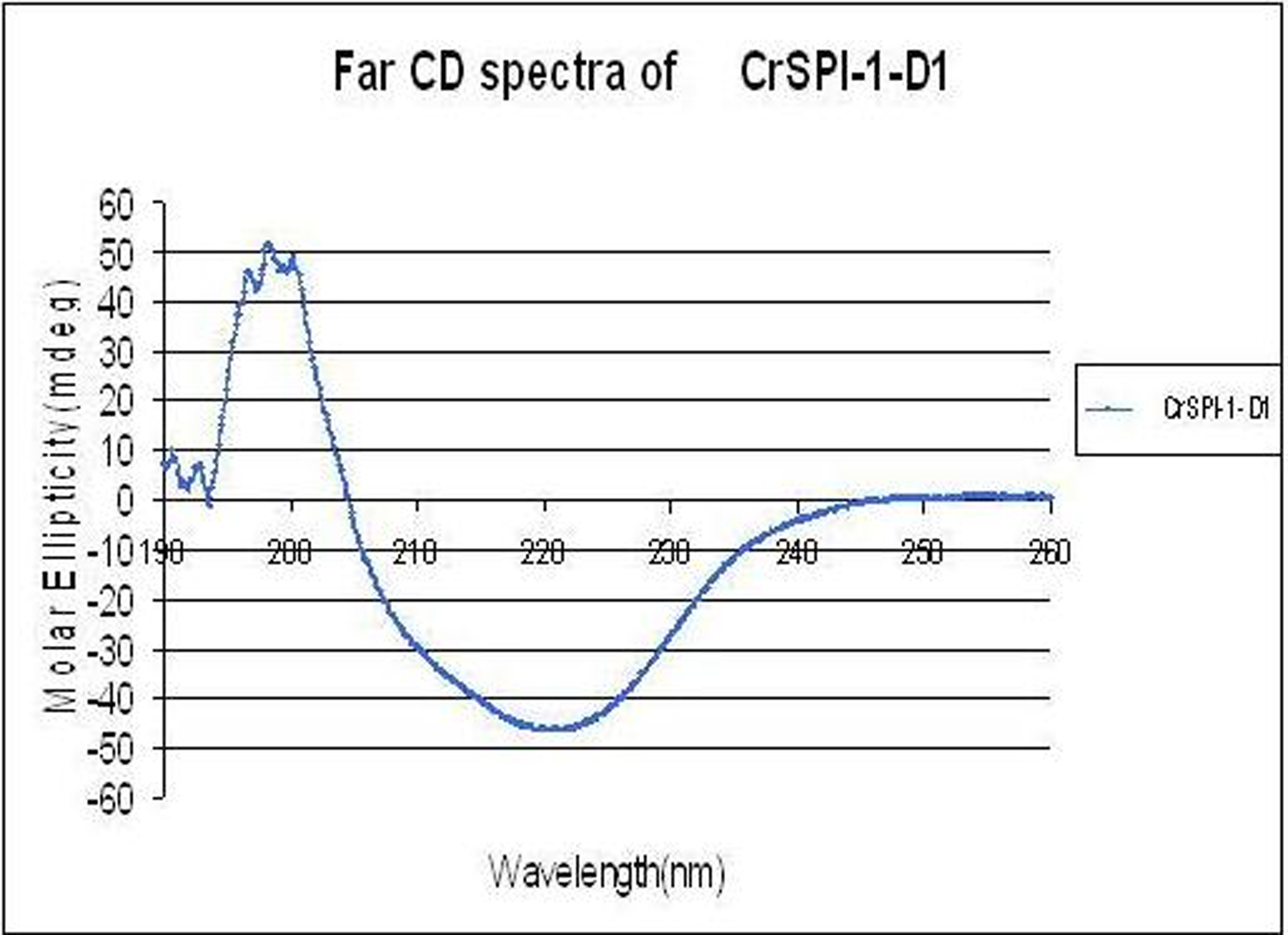

Supplement: Figure S2 — CD spectroscopy profile of reverse phase HPLC purified CrSPI-1-D1. Far-UV CD spectra (260–190 nm) of CrSPI-1-D1 dissolved in 20 mM Tris-HCl buffer (pH 7.4) at a 30 μM protein concentration were collected using a Jasco J-810 spectropolarimeter (Easton, MD). All measurements were carried out at room temperature using 0.1-cm path length cuvettes with a scan speed of 50 nm/min, a resolution of 0.2 nm, and a bandwidth of 2 nm. The CD spectrum of the tetra mutant of CrSPI-1-D1 indicated that it assumed an α/β structure. (TIF) [file pone.0015258.s004.tif]

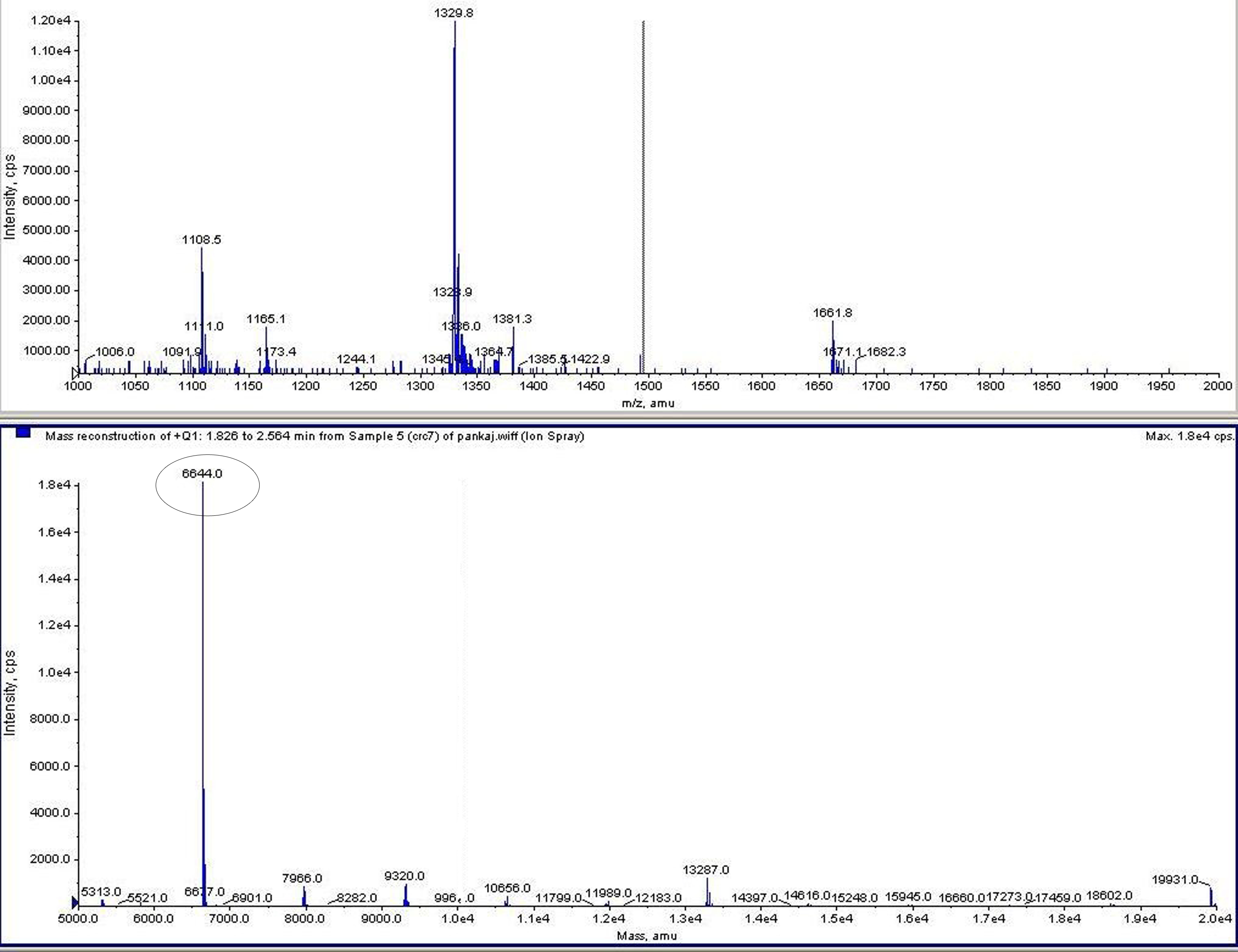

Supplement: Figure S3 — ESI/MS profile of reverse phase HPLC purified CrSPI-1-D1. The spectrum shows a series of multiply charged ions, corresponding to the correct molecular mass of 6644± 0.22 Da. The purity and mass of all mutant proteins of CrSPI-1-D1 were determined by electro spray ionization mass spectrometry using an API 300 liquid chromatography tandem mass spectrometry system (PerkinElmer Life Sciences Sciex, Selton, CT). (TIF) [file pone.0015258.s005.tif]

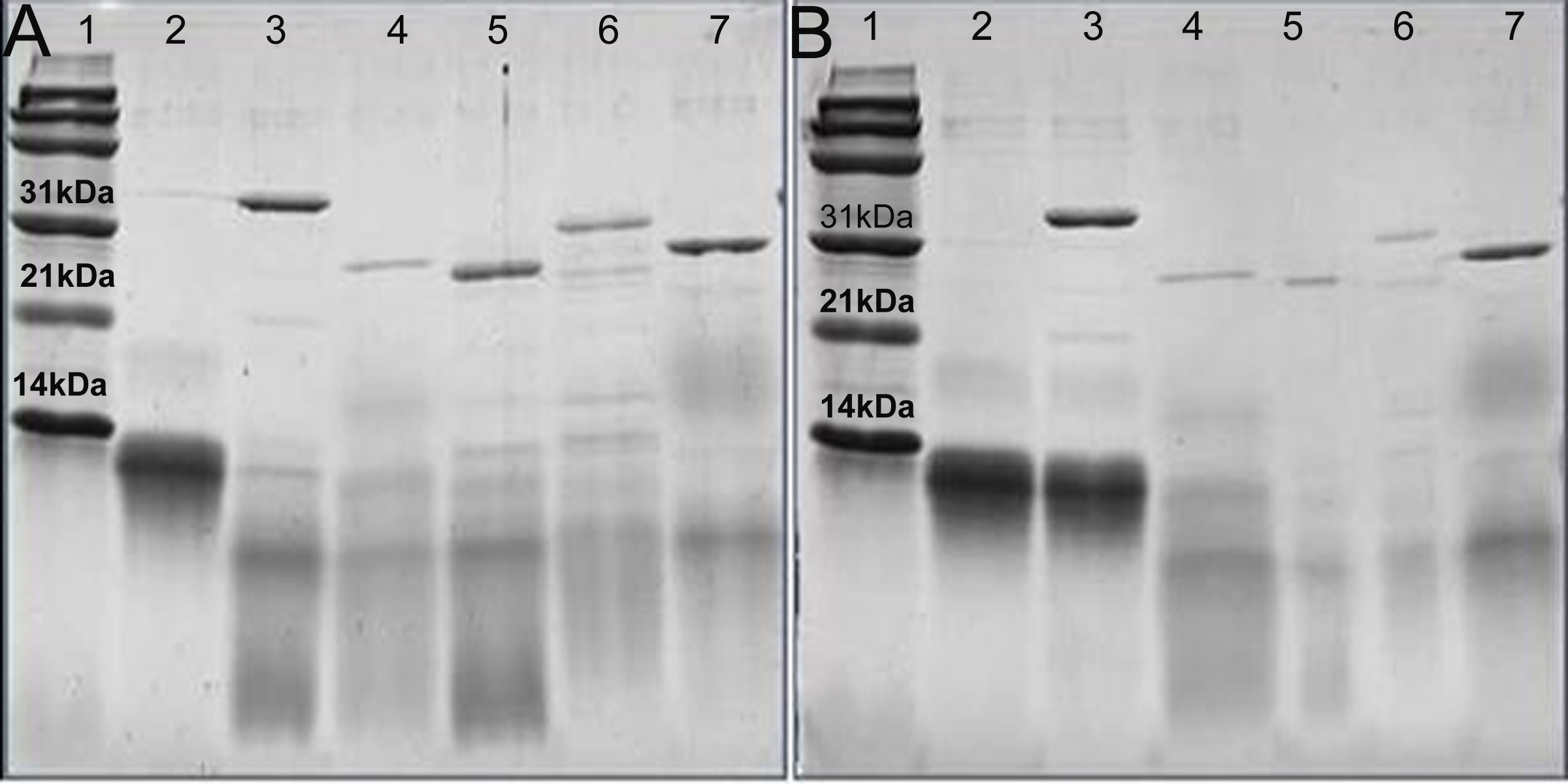

Supplement: Figure S4 — The specificity of CrSPI-1-D1 tetra mutant for thrombin ascertained by comparison with other proteases. SDS-PAGE analysis for the interaction of CrSPI-1-D1 wild type and tetra mutant with different proteases. A ) Lane 1 protein marker; Lane 2 CrSPI-1-D1 alone and Lane 3-7 CrSPI-1-D1 wild type incubated with human α-thrombin, chymotrypsin, trypsin, elastase and subtilisin, respectively, for 37°C for 30 minutes. B ) Lane 1 protein marker; Lane 2 T4A, Y5K, K6H, P7R CrSPI-1-D1 alone and Lane 3-7 T4A,Y5K, K6H, P7R CrSPI-1-D1 incubated with human α-thrombin, chymotrypsin, trypsin, elastase and subtilisin, respectively, for 37°C for 30 minutes. (TIF) [file pone.0015258.s006.tif]
